# Does SDG 3 have an adequate theory of change for improving health systems performance?

**DOI:** 10.7189/jogh.07.010302

**Published:** 2017-06

**Authors:** Gabriel Seidman

**Affiliations:** Harvard TH Chan School of Public Health, Boston, Massachusetts, USA

Given the importance of Sustainable Development Goal 3 (SDG 3) setting national agendas and policies to improve public health, this article examines whether SDG 3 and its associated indicators have an adequate theory of change for improving health systems performance. To do so, this article maps all SDG 3 indicators to a prominent health systems framework. The analysis reveals that SDG 3 tracks four input indicators, 15 output / outcome indicators, and 18 impact indicators. Unlike the Millennium Development Goals (MDGs), SDG 3 tracks population health across a wide array of disease areas. However, SDG 3 has several limitations in its approach to improving health systems performance. It does not track primary health care inputs, financial risk protection, or user satisfaction with the health system, and it does not provide a comprehensive approach to prevent, diagnose, treat, and manage any disease. Future directions for research include conducting a similar mapping for other SDGs and documenting early country experiences implementing SDG 3 given these challenges.

## INTRODUCTION

Sustainable Development Goal (SDG) 3 aims to “ensure healthy lives and promote well–being for all at all ages” [1]. Unlike the Millennium Development Goals (MDGs), SDG 3 takes a comprehensive view of health and well–being by expanding its focus beyond a core set of diseases. Given the global prominence of the SDGs for driving the development agenda, it is important to consider whether SDG 3 and the indicators it tracks are well–designed to achieve this intended goal.

In order to examine whether SDG 3 can actually help achieve this goal, this article considers whether it has an adequate theory of change (ToC) for improving health systems performance. Such an analysis rests on two core assumptions: 1) in order to achieve the SDG 3 goal, one must improve health systems performance, and 2) in order to achieve this goal, the approach must have a strong underlying ToC. Each assumption is considered below.

Since the launch of the MDGs, experience has shown that without improvements in health systems performance, progress on the MDGS was both limited and potentially unsustainable [2]. Bottlenecks in the health system limited nations’ ability to achieve progress on combatting specific diseases. In addition, theoretical and empirical work has argued that providing services which are not only clinically effective but also affordable and acceptable has intrinsic and instrumental value. Recognizing the importance of overall health systems performance, numerous organizations including WHO, the World Bank, Global Fund, and GAVI have focused on health systems strengthening (HSS) as an important component of public health programming. Therefore, since SDG 3 aims to improve both health and well–being for all populations in a sustainable way, achieving this goal will likely require broad improvements in health systems performance.

With regards to the second assumption, theories of change (ToC) are standard practice in public health and development [3,4]. They help guide priority–setting, decision–making, monitoring and evaluation, budgeting, and resource allocation, among other activities. A strong ToC can ensure that all stakeholders work toward the same goal(s). The SDGs aim to improve both the coherence of development policies and their implementation at the national level, and the United Nations has offered formal guidance on ways that nations can integrate and tailor the SDGs into their national policies [5]. This guidance explicitly advocates for horizontal policy coherence (ie, coherence across different programs and sectors), vertical policy coherence (ie, coherence between different stakeholders), and linking national policies based on the SDGs to budgets [6]. Given that the SDGs aim to improve policy coherence and drive implementation at the national level, it is instructive to consider whether they have internal coherence and a strong underlying logic themselves. The ToC approach provides a useful approach to explore this question. If the inputs and outputs tracked under SDG 3 have clear linkages to improving its impact indicators, then working toward SDG 3 will allow countries to pursue a comprehensive program for improving health systems performance. On the other hand, if the inputs and outputs tracked do not link to each other or do not have logical connections to impact indicators, then aiming to improve all indicators under SDG 3 could lead to a haphazard and uncoordinated set of public health programs.

## ANALYTIC APPROACH

To address this question, this analysis maps all indicators for SDG 3 as inputs, outputs/outcomes, or impacts, using the formal definition of each [7]:

- **Inputs:** “The human financial, and community resources a program has available toward [implementing a program]”
- **Outputs:** “Direct products of program activities”
- **Outcomes:** “Specific changes in…behavior, knowledge, skills, status, and level of functioning”
- **Impacts:** “The fundamental intended or unintended change occurring in organizations, communities, or systems”

All indicators are drawn from the official list available from the UN Statistics Division and associated metadata [1]. The 16 tracer indicators proposed by the Inter–agency and Expert Group (IAEG) on SDGs for indicator 3.8.1 (coverage of essential health services) were also included for analysis [8]. This analysis refers to these indicators as UHC tracer indicators.

Health systems theory formally defines the three intended impacts of a health system as population health status, financial risk protection, and user satisfaction with the system [9–11]. Working backward from this definition, outputs/outcomes should refer to the results of activities or changes in the population which can ultimately affect at least one of these three impacts, and inputs should refer to any resources in the system directed toward changing outputs, outcomes, or impacts.

**Figure 1:**
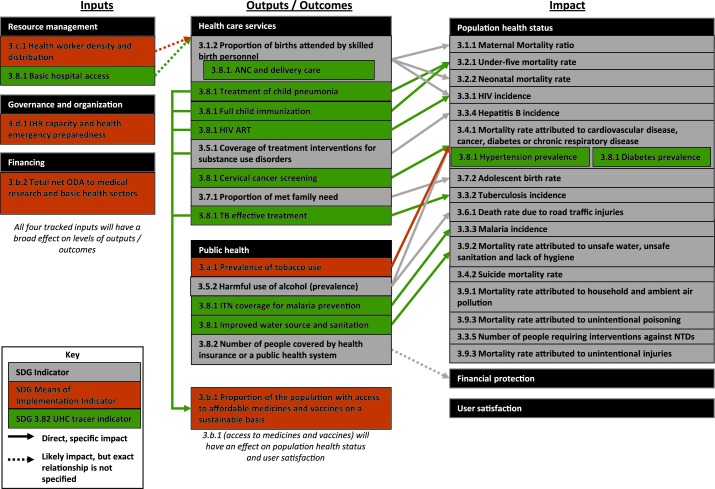
Mapping of SDG 3 indicators to a health systems framework.

In order to map SDG 3 indicators, this analysis uses a health systems framework which has a structure very similar to that of a standard ToC [9]. This framework clearly maps to inputs, outputs/outcomes, and impacts, and it further breaks down each of these three areas into relevant sub–categories (**Figure 1**). Of course, many other frameworks for health systems exist, such as the WHO Building Blocks and the Flagship Framework, and one could also conduct this mapping exercise using those frameworks. However, these two frameworks do not fit as closely with a standard ToC; the Building Blocks framework does not necessarily explain how different building blocks relate to each other, and the Flagship Framework does not explicitly consider inputs to the health system [12].

## RESULTS OF MAPPING SDG INDICATORS TO A HEALTH SYSTEMS THEORY OF CHANGE

This section summarizes the key results of mapping SDG 3 indicators to a ToC. Of the 16 UHC tracer indicators, the analysis excluded five that were duplicative with SDG tracer indicators (excluded UHC tracer indicators, and the SDG indicators that they duplicated: family planning coverage [3.7.1], Tobacco, non–use [3.a.1], Health worker density [3.c.1], Access to essential medicines [3.b.1], and Health security: IHR compliance [3.d.1]). See **Figure 1** for a schematic of the full results of this mapping exercise.

## INPUTS (AND MEANS OF IMPLEMENTATION TARGETS)

SDG targets are divided into Means of Implementation (MoI) targets and other targets [13]. MoI targets are meant to summarize the resources needed to achieve all other targets. SDG 3 has five MoI targets: A) prevalence of tobacco use, B.1) proportion of the population with access to affordable medicines and vaccines, B.2) total net ODA to medical research and basic health sectors, C) health worker density and distribution, and D) IHR capacity and emergency health preparedness. Three of these indicators (total net ODA, health worker density and distribution, and IHR capacity) serve as inputs to the health system, whereas the other two are indicators/outcomes. The analysis also classifies one UHC tracer indicator, basic hospital access, as a health systems input.

**Figure:**
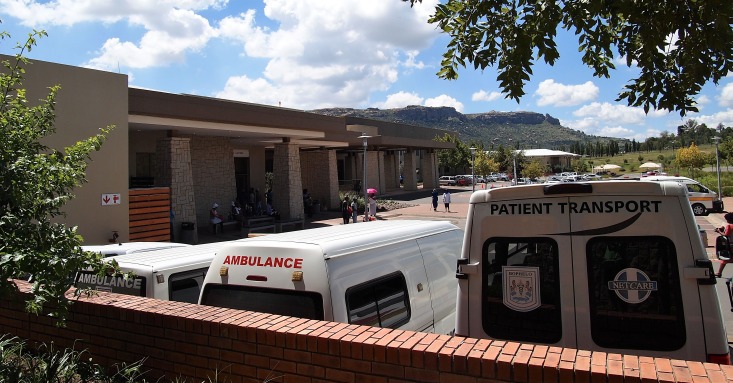
Photo: The Queen ‘Mamohato Memorial Hospital in Lesotho.Experience with this hospital highlighted problems of investing in hospital care without a strong primary health care system. Photo from the collection of Patrick Smith, used with permission. More at: A dangerous diversion: Will the IFC's flagship health PPP bankrupt Lesotho's Ministry of Health? OXFAM Briefing Note, 7 April 2014.

The four indicators classified as inputs fall across the categories of financing, resource management, and governance and organization. With regards to financing, Indicator B.2, total ODA for health, can have an impact on health systems outputs, but there is no clear linkage between this indicator and any specific outputs or impacts tracked by SDG 3. It is not even clear what improvement on this indicator would look like. While an increase in ODA may signify increasing expenditure on health, it does not take into account government and out–of–pocket spending on health, and it may also cause or exacerbate issues with donor dependency in low– and middle–income countries (LMICs). Further, given the variation in efficiency of health spending across countries, an increase in total ODA may not necessarily represent any changes to health systems outputs.

The two resource management indicators, total health workforce and basic hospital access, will impact the availability of health care services and associated outputs. Indicator C, total health workforce, currently measures total physicians and nurses/midwives per capita and will likely expand to include dentists, pharmacists, and possibly other health personnel [14]. The one governance and organization indicator, International Health Regulation (IHR) compliance, focuses on key competencies designed to “prevent, protect against, control and provide a public health response to the international spread of disease” [15].

The fact that two MoI indicators represent outcomes, rather than inputs into the system, suggests that measuring them will not necessarily help countries identify the root causes of problems in the health system or ways to address these issues. Indicator A, prevalence of tobacco use, is meant to measure implementation of the WHO Framework Convention on Tobacco Control, but is actually an intended outcome of this regulation, not a measurement of implementation itself. As of the end of 2016, the UN had not released a final definition of metadata for Indicator B.1 (proportion of the population with access to medicines and vaccines). However, the wording of this indicator suggests that it does not actually capture an input, such as the (per–capita) number of medicines in the country or existence of a national essential medicines list, but rather what percentage of the population gets access to these medicines. Therefore, poor performance on this indicator will not necessarily indicate a shortage of medicines, since populations might lack access to medicines for other reasons (eg, physical distance to a health center, poor supply chain management). Further, four of the 16 UHC tracer indicators monitor the coverage for medicines and vaccines, and may therefore duplicate Indicator B.1.

Many of the outputs / outcomes and impact indicators discussed below have no MoI indicators which directly precede them or influence their progress. There are no input indicators which measure access to primary health care, community–based health services, or health education.

## OUTPUTS/OUTCOMES

Five SDG indicators and eight UHC tracer indicators classify as health systems outputs or outcomes (in addition to the two MoI indicators mentioned earlier). There is at least one indicator that tracks an output related to each Millennium Development Goal (MDG) priority disease (HIV, malaria, maternal health, newborn / child health), as well as TB, substance use disorders, cervical cancer, family planning, tobacco use, alcohol use, and water/sanitation. These indicators are split across health care services and public health. All disease–related outputs link to at least one health systems impact indicator, suggesting that there is a clear and logical linkage between improvements on health systems outputs and health systems impacts.

Indicator 3.8.2, the number of people covered by health insurance, does not link to any impact indicator tracking financing risk protection (such as the percent of the population experiencing catastrophic or impoverishing health expenditures).

The MoI indicator tracking proportion of population with access to medicines and vaccines (B.1) will of course change impact indicators, but it is impossible to assess how it will do so until the UN releases the tracer medicines which will make up this indicator.

## IMPACTS

All impact indicators measure population health status across a wide variety of disease areas. Some measure disease transmission rates (eg, HIV incidence, TB incidence), whereas many others measure mortality rates. There are no indicators to track financial risk protection or user satisfaction.

Although every output indicator links to an impact indicator, not every impact indicator has a preceding output indicator. Indeed, of 18 impact indicators, five do not have a preceding output indicator (mortality due to suicide, air pollution, unintentional poisoning, neglected tropical diseases, and unintentional injuries). This suggests that the SDGs do not give clear guidance on how to address one–third of the impacts targeted. Of the 12 impact indicators which do have a preceding output, 11 have a preceding output indicator from either health care services or public health, but not from both. The one impact indicator which has preceding outputs from both health care services and public health is 3.4.1 (mortality from select NCDs), and the preceding outputs (cervical cancer screening, prevalence of tobacco use, and harmful use of alcohol) actually address very different diseases.

## SELECT LIMITATIONS OF SDG 3 AND POTENTIAL IMPLICATIONS

The results of this mapping exercise have important implications for public health programming and policies. Unlike the MDGs, SDG 3 clearly takes a comprehensive view of the potential epidemiological challenges that a country may face. However, SDG 3 fails to take a holistic view of the health system, its goals, and the resources /activities needed to achieve these goals. In particular, SDG 3 has three limitations for guiding policy and practice to improve health systems performance. See **Table 1** for a summary of the limitations of SDG 3 and potential implications.

**Table 1:** Select limitations of SDG 3 identified by this analysis and potential implications

Limitations of SDG 3	Potential implications for policy and practice
SDG 3 does not systematically track indicators related to primary health care, which can serve as the foundation for a strong health system.	Policymakers and practitioners should consider how to integrate a PHC–based approach which can effectively and efficiently improve health systems performance into the SDG indicators in their specific contexts.
SDG 3 does not provide guidance on how to systematically prevent, diagnose, treat, or manage and given disease.	Policymakers and practitioners should formulate and implement holistic approaches to addressing the highest burden diseases in their specific context, while specifically considering how to integrate these efforts into the health system, including PHC–based approaches.
SDG 3 does not track impacts related to financial risk protection or user satisfaction with the health system.	Policymakers and practitioners should include indicators on financial risk protection and user satisfaction in their monitoring and evaluation of the health system and design health systems components, such as insurance schemes and essential medicines packages, with these in mind.

1 **Few indicators track the status of the primary health care (PHC) system.** A strong PHC system can serve as the basis for achieving universal health coverage and a stronger health system overall [16]. Given that PHC can address 90% of health care demands, and many “good buys” for combatting diseases can be integrated into PHC systems, prioritizing hospital access over PHC access can lead to inefficiency and misallocation of resources in the health system [10,17]. However, SDG 3 places very little focus on PHC. The two indicators for resource management – health worker density and hospital access – neglect key health systems inputs at the PHC and community levels, such as access to a PHC clinic, the availability of essential medicines at these clinics, health education, and the ratio of lay health workers such as community health workers per population. Given the importance of PHC for improving population health and creating the foundation for a strong health system, policymakers and practitioners should consider how to integrate a PHC approach into achieving SDG 3.
2 **There is no comprehensive approach to prevent, diagnose, manage, and treat any disease.** As mentioned earlier, an impact indicator for a specific disease links to a preceding output indicator either from health care services or public health, but never both. This structure suggests that, while SDG 3 identifies targeted interventions that can address many of its priority diseases, it does not promote a comprehensive approach to preventing, diagnosing, treating, and managing any given disease. The output indicators also do not track certain key health behaviors which can impact population health through disease prevention, such as condom usage and physical exercise. (SDG 3 also does not include any indicators on nutrition, but SDG 2 covers these.) Further, of the five impact indicators which have no preceding outputs, many can be addressed through environmental health or other programs. Policymakers and practitioners should recognize that the disease–specific guidance in SDG 3 is only very summary, and that an internal ToC is likely needed for improving population health for each disease. Following from the point about PHC, policy and practice should also consider how integrated prevention, diagnosis, treatment, and management can occur at the PHC level.
3 **The indicators do not track impacts related to financial risk protection or user satisfaction with health services.** As already discussed, ignoring these indicators has significant implications for the functioning of country’s health system. Failing to protect individuals against financial risk from health expenditures can negatively impact people’s access to care as well as the non–health aspects of their lives [18]. Similarly, a patient’s satisfaction with services and the overall responsiveness of the health system to the patient’s needs can impact patient well–being and future interactions with the health system [19]. Policymakers and practitioners should take into account the effect that providing services to improve population health will have on patient’s financial status and satisfaction with the health system, as well as the linkages between these impacts and population health.

Overall, this lack of a systems–wide approach for improving public health could lead to significant challenges for countries aiming to implement SDG 3. In particular, it could limit the overall improvements to health systems performance because it could promote an uncoordinated approach to improving health, especially without a focus on PHC.

## LIMITATIONS OF THIS APPROACH

This analytic approach has several limitations. As mentioned earlier, many health systems frameworks exist, and this mapping exercise could use other frameworks which might lead to different conclusions. In addition, as with any ToC, this approach presents a highly linear way to understand SDG 3 and its underlying logic. Of course, as the field of systems thinking has revealed, changes to complex systems can have unpredictable and multi–directional results, and this analytic approach does not reflect that complexity [20]. This approach does not map the linkages between impact indicators (eg, a change in HIV incidence could affect other impact indicators such as TB incidence and under–five mortality). Nonetheless, clearly laying out the first–order linkages between different SDG 3 indicators at least gives a working model for understanding if this model provides a logical and robust approach to improving public health more broadly.

## CONCLUSION AND FUTURE DIRECTIONS

This article likely represents the first attempt to map SDG 3 indicators to a ToC for improving health systems performance. This mapping highlights several challenges with the structure of SDG 3, namely the lack of a holistic approach to improving health systems performance. Given the novelty of the SDGs, it is still too early to evaluate the impact of this potential shortcoming. However, these findings point to two key next steps for future investigation. First, researchers can systematically map the indicators for the other SDGs in a similar way and link the ToCs from different SDGs to each other. Doing so will help identify similar challenges for other SDGs, as well as potential linkages between the SDGs. Second, researchers, practitioners, and policymakers should document early experiences trying to implement SDG 3 to determine whether countries recognize these implicit limitations and, if so, how they are responding to them.
